# Neuromodulation and rehabilitation of post-stroke cognitive impairment: challenges and prospects

**DOI:** 10.3389/fpsyt.2026.1780907

**Published:** 2026-02-26

**Authors:** Wenya Shang, Bareun Choi, Qingyang Zhan, Jinglei Wu, Dongsheng Xu

**Affiliations:** 1 School of Rehabilitation Science, Shanghai University of Traditional Chinese Medicine, Shanghai, China; 2 Engineering Research Center of Traditional Chinese Medicine Intelligent Rehabilitation, Ministry of Education, Shanghai, China; 3 Institute of Rehabilitation Medicine, Shanghai University of Traditional Chinese Medicine, Shanghai, China; 4 Department of Rehabilitation Medicine, Yueyang Hospital of Integrated Traditional Chinese and Western Medicine, Shanghai University of Traditional Chinese Medicine, Shanghai, China

**Keywords:** brain-computer interface, genetic-based neuromodulation, neuroimaging, neuromodulation, post-stroke cognitive impairment, transcranial electrical stimulation, transcranial magnetic stimulation

## Abstract

It is essential to recognize the significant daily impact that post-stroke cognitive impairment (PSCI) has on patients and their families. Neuromodulation strategies have been increasingly applied in the clinical management of PSCI. This review outlines the mechanisms and brain function detection approaches through which neuromodulation promotes cognitive enhancement in stroke patients. For cognitive recovery, transcranial magnetic stimulation, transcranial electrical stimulation, vagus nerve stimulation, and brain-computer interfaces have shown promising results in clinical and preclinical studies. However, their efficacy remains unproven in large-scale pivotal trials. Preliminary clinical trials have shown that photobiomodulation enhances cognitive performance, but further investigation is required into the issue of skull attenuation of light. Transcranial ultrasound stimulation, a novel technology that overcomes the limitation of requiring deep electrode implantation for focal deep brain stimulation, still lacks scientific evidence. Chemogenetics and optogenetics provide methods for monitoring, disrupting, and regulating neural circuits after a stroke. To enhance the effectiveness of neuromodulation, it is recommended to implement multi-target stimulation, strengthen active participation in rehabilitation, and leverage cognitive-motor interactions to promote holistic recovery after stroke. Finally, we propose that neuromodulation will evolve toward brain-machine interaction neuromodulation, using artificial intelligence to develop a closed-loop strategy encompassing stimulation, detection, optimization, and re-stimulation.

## 1 Introduction

Stroke is a prevalent disorder that can result in neurological deficits due to disturbance in cerebral blood circulation, with approximately one-third of global healthcare expenditures allocated to its management (1, 2). Within one year of a stroke, approximately 60% of patients develop cognitive impairment (3). Post-stroke cognitive impairment (PSCI) is a clinical syndrome induced by stroke, characterized by impairments in cognitive functions, including attention, memory, language, executive function, and visuospatial ability. In patients with stroke, early interventions can help reverse cognitive impairment, but the insidious onset of the condition leads to nearly a third developing dementia within five years (4). Over 80% of stroke patients experience concurrent cognitive and motor impairments. This dual dysfunction severely affects patients’ quality of life and imposes a substantial socioeconomic burden. When stroke patients experience impairments in attention, executive function, or working memory, their balance, movement initiation, and motor learning are often adversely affected. Conversely, cognitive enhancement can facilitate motor recovery and thereby promote overall functional rehabilitation (5). This emphasizes the importance of the bidirectional facilitation between cognition and motor function.

**Figure 1:**
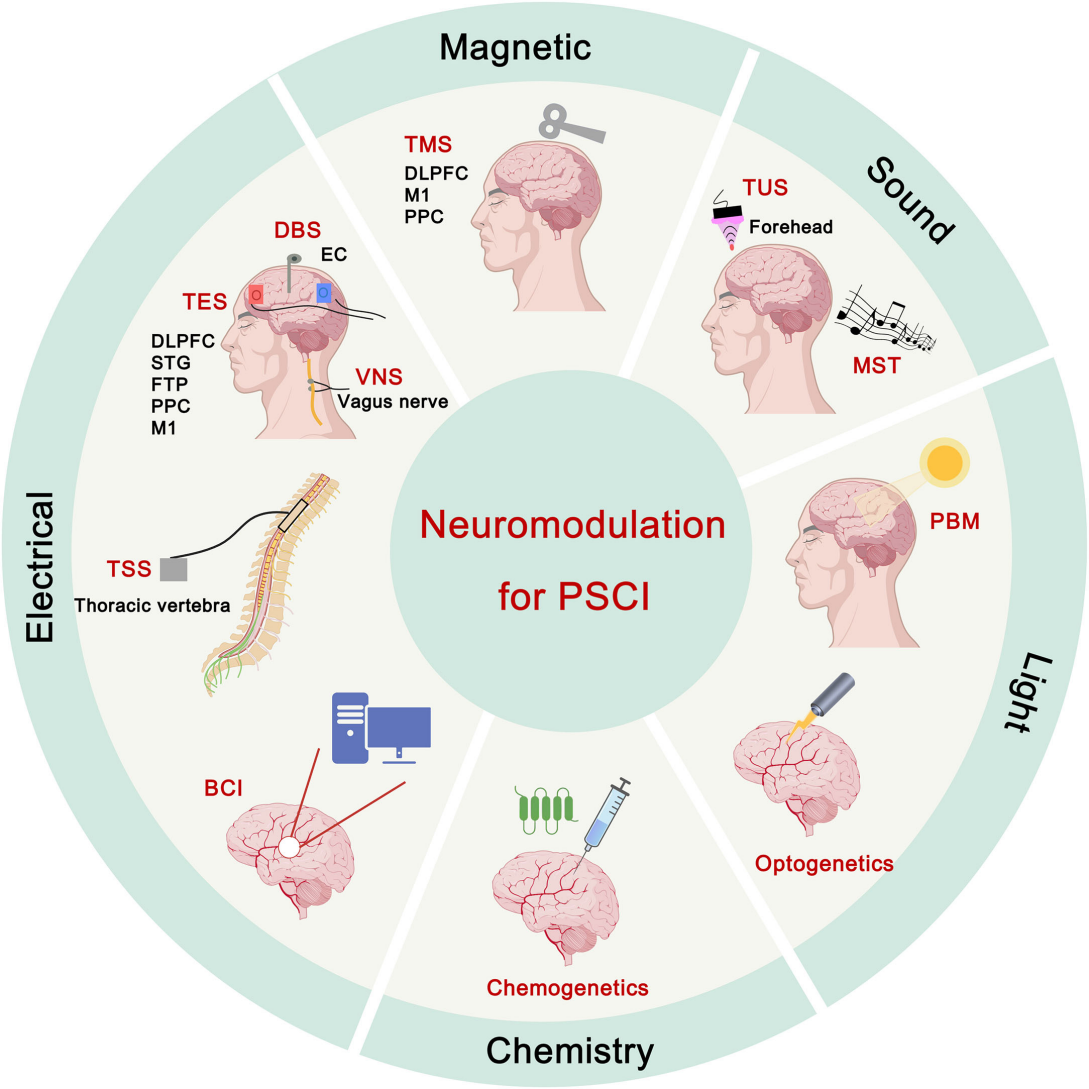
Schematic diagram of neuromodulation strategies and common targets for post-stroke cognitive impairment (PSCI). Neuromodulation strategies, including genetic and non-genetic techniques, primarily employ electrical, magnetic, light, sound, and chemical modalities to restore neural circuitry damaged by stroke. PSCI, post-stroke cognitive impairment; TMS, transcranial magnetic stimulation; DBS, deep brain stimulation; TES, transcranial electrical stimulation; VNS, vagus nerve stimulation; TSS, transcutaneous spinal stimulation; BCI, brain-computer interface; PBM, photobiomodulation; MST, music-supported therapy; TUS, transcranial ultrasound stimulation; DLPFC, dorsolateral prefrontal cortex; M1, primary motor cortex; PPC, posterior parietal cortex; EC, entorhinal cortex; STG, superior temporal gyrus; FTP, fronto-temporal region. Figure created using BioGDP.com (7).

The mechanisms underlying PSCI extends beyond structural damage at the lesion site; it may also involve disruptions in brain network connectivity or even whole-brain functional connectivity. This emphasizes the critical role of neuromodulation strategies. Neuromodulation strategies have been demonstrated to modulate neuronal activity, regulate neuroplasticity, and repair damaged neural circuits through various forms of stimulation, including electrical, magnetic, and other physical or chemical methods (6). This process helps modulate relevant brain functions to improve cognitive function in patients. In recent decades, neuromodulation strategies have shown promise in the regulation of cognitive brain function, including transcranial magnetic stimulation (TMS), transcranial electrical stimulation (TES), vagus nerve stimulation (VNS), photobiomodulation (PBM), transcranial ultrasound stimulation (TUS), brain-computer interface (BCI), deep brain stimulation (DBS), transcutaneous spinal stimulation (TSS), music-supported therapy (MST), and genetic-based neuromodulation (**Figure 1**).

This review outlines the mechanisms and brain function detection approaches through which neuromodulation promotes cognitive enhancement in stroke patients. In this context, we critically evaluate the efficacy of neuromodulation strategies in both preclinical and clinical settings. We also discuss the future prospects of neuromodulation for PSCI from the perspectives of multi-target and multimodal strategies, as well as motivation-enhanced holistic rehabilitation approaches. It highlights that non-invasive, home-based, multi-target, multimodal, closed-loop, and intelligent brain-machine interaction neuromodulation represents an increasingly vital and promising area of research.

## 2 Search strategy

This review cited studies through searches of PubMed, Embase, Web of Science, and Scopus, including publications up to and including 2025. Only English-language studies were included. Our search strategy using the keywords extracted from the MeSH and Emtree databases, including “stroke,” “cognition,” “neuromodulation”, “transcranial magnetic stimulation,” “transcranial electrical stimulation,” “vagus nerve stimulation,” “photobiomodulation,” “transcranial ultrasound stimulation,” “brain-computer interface,” “deep brain stimulation,” “transcutaneous spinal stimulation,” “music-supported therapy,” “optogenetics,” and “chemogenetics.” The studies were screened based on their titles and abstracts, and those meeting the inclusion criteria were subjected to further analysis. Furthermore, searches were conducted using these keywords on https://clinicaltrials.gov/.

## 3 Multiscale neuromodulatory mechanisms promoting recovery of cognitive function

### 3.1 Neuromodulatory mechanisms at the microscale

The brain is a highly intricate network of anatomically interconnected neural components that interact continuously. A critical challenge in neuroscience is to understand how macroscale brain dynamics are shaped by the underlying microscale mechanisms (8). At the microscale, neuroplasticity relies on activity-dependent mechanisms that modulate the strength and efficiency of synaptic transmission. Key processes involved neurotransmitter release, calcium ion channels, N-methyl-D-aspartate (NMDA) receptors, α-amino-3-hydroxy-5-methyl-4-isoxazole propionic acid (AMPA) receptors, and intracellular signaling pathways (9). High-frequency activation of AMPAR induces Ca^2+^ influx into the postsynaptic neuron via NMDAR and L-type Ca^2+^ channels. This elevation in intracellular Ca^2+^ concentration resulting in the integration of AMPARs into the postsynaptic membrane, thereby improving postsynaptic responses. The aforementioned process of synaptic strengthening is called long-term potentiation (LTP), whereas the reduction in synaptic strength through AMPAR endocytosis is called long-term depression (LTD) (10). Abnormal changes in LTP and LTD lead to reduced synaptic efficiency, impairing the brain’s capacity to receive and process external information, ultimately manifests as cognitive impairments. Decades of neurobiological studies have demonstrated that neuromodulation strategies can improve cognitive function in PSCI by influencing cortical microstructural properties, including neurotransmitter levels, cytoarchitecture, dendritic cell type size, density and distribution, synaptic structure, and gene expression (8).

TMS enhanced synaptic density and active zone length, reduced synaptic cleft width, and upregulated the levels of PSD95, SYN, and brain-derived neurotrophic factor (BDNF) in the hippocampus (11). At the functional level, High-frequency repetitive TMS (HF-rTMS) enhanced LTP at CA3–CA1 synapses and synaptic plasticity by modulating the vascular endothelial growth factor (VEGF) and BDNF–NMDAR signaling pathways in the hippocampus, thereby facilitating spatial learning capacity in PSCI rats (12). Furthermore, rTMS has been shown to regulate the expression of genes related to glycinergic, glutamatergic, and GABAergic neurotransmission systems in cerebellum and brainstem, suggesting that it may modulate neuronal activity and synaptic plasticity by altering neurotransmitter levels (13). GABAergic activity plays a crucial role in shaping induced and evoked cortical oscillations, particularly in enhancing theta and gamma oscillations following a stroke (14). Wu et al. (15) suggested that intermittent theta-burst stimulation (iTBS) enhances hippocampus-dependent memory, potentially through augmented theta oscillations and changes in extracellular GABA and glutamate levels in the dorsal hippocampus. Similarly, the combination of transcranial direct current stimulation (tDCS) and donepezil has been shown to mitigate or delay cognitive impairment, enhance memory recall, and elevate acetylcholine levels in the cerebral cortex of stroke patients (16). VNS may promote norepinephrine release, thereby enhancing spatial and fear memory following cerebral ischemia (17).

### 3.2 Neuromodulatory mechanisms at the mesoscale

Mesoscale mechanisms are processes that occur within neural circuits, which consist of connections between various types of neurons across brain regions. iTBS-induced cognitive improvement was related to the activation of the dorsolateral prefrontal cortex (DLPFC) and other distant regions, thereby improving assessment, decision-making capacities and cognitive control. In contrast, tDCS-induced cognitive improvement was associated with the activation of the frontopolar cortex, thereby enhancing valuation, motivation, and decisional substructures in PSCI patients (18). Furthermore, activation in the left superior temporal cortex, as measured by functional near-infrared spectroscopy (fNIRS), is also one of the mechanisms underlying tDCS treatment for PSCI (19).

Brain oscillations are rhythmic patterns of neural activity that emerges from interactions between intrinsic neuronal properties and the dynamics of interconnected neural networks (20). At the mesoscale, neuronal death in brain regions disrupts signal transmission within neural circuits, leading to abnormal structural or functional connectivity and neural oscillations. In a pilot trial, researchers found that theta-frequency rhythmic TMS targeting the parietal cortex improved performance on an auditory Working memory (WM) task by enhancing the oscillatory entrainment of frontoparietal theta rhythms (21). Song et al. (22) indicated that HF-rTMS reduces gamma oscillatory activity, enhances theta- and alpha-band functional connectivity within the DLPFC, and facilitates cognitive recovery in patients with PSCI. The application of transcranial alternating current stimulation (tACS), which involves the induction of a rhythmic oscillating flow of exogenous current associated with the endogenous brain oscillation models, can induce and affect endogenous brain oscillations that are frequency specific. This process is known as “neuronal entrainment” (23). Applying high-definition (HD)-tACS at alpha frequency to the unaffected hemisphere may enhance spatial attention in stroke patients by supporting the activity of unilateral alpha oscillations (24). Similarly, studies have shown that transcranial random noise stimulation (tRNS) regulates theta and gamma activity during the WM encoding phase and is more effective than tDCS in improving WM in healthy participants (25). Furthermore, Zheng et al. (26) demonstrated that 40 Hz light flicker alleviates the reduction of low gamma oscillations in the hippocampal CA1 region following cerebral ischemia and facilitates cognitive recovery. Mechanistically, it improves RGS12-regulated, CA3-CA1 presynaptic N-type calcium channel-dependent short-term synaptic plasticity and promotes postsynaptic LTP a model of cerebral ischemia. Overall, entrainment through rhythmic stimulation near or at the brain’s natural oscillations can facilitate more precise and personalized neuromodulation.

### 3.3 Neuromodulatory mechanisms at the macroscale

Evidence from preclinical and clinical neuroimaging studies indicates that cognitive impairment after stroke results from alterations in whole-brain networks rather than the loss of a single component (27). At the macroscale, neuromodulation facilitates whole-brain functional network reorganization, ultimately improving the overall function and behavior of stroke patients. Targeting specific cerebral regions with TMS in PSCI patients affects not only the directly stimulated areas but also distant brain regions through neural network interactions, resulting in lasting changes in inter-brain connectivity that underlie TMS-induced cognitive improvements. After rTMS treatment, patients with PSCI exhibited higher fractional amplitude of low-frequency fluctuation (fALFF) levels in the inferior frontal gyrus, superior temporal gyrus, and parahippocampal gyrus, and lower fALFF levels in the middle frontal gyrus, middle temporal gyrus, and fusiform gyrus. Additionally, rTMS enhanced functional connectivity (FC) between DLPFC and toprecuneus, marginal gyrus, inferior temporal gyrus, and middle and inferior frontal gyrus, while reducing FC between DLPFC and thalamus and middle temporal gyrus (28). Moreover, VNS is more effective in enhancing executive functions owing to elevated activity in the brainstem nuclei and nucleus of the solitary tract (NTS). Specifically, projections from the NTS to the locus coeruleus and dorsal raphe nucleus facilitate the circulation of monoaminergic neurotransmitters. Norepinephrine circulation may enhance functional connectivity between the prefrontal cortex (PFC), hippocampus, and amygdala, thereby promoting the formation of episodic memories, improving attention and alertness, and modulating emotional responses (29).

**Figure 2:**
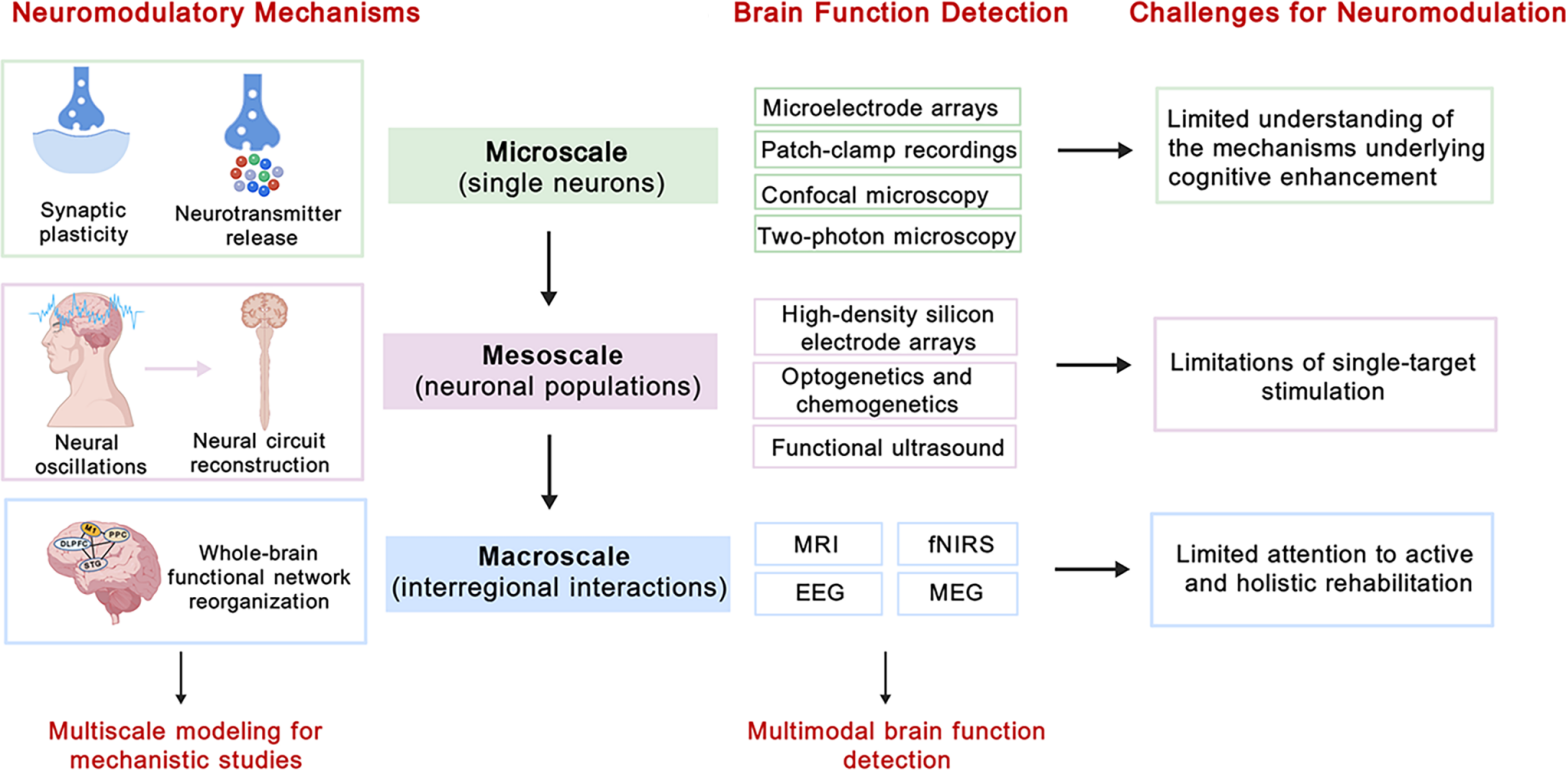
Mechanisms, brain function detection approaches, and challenges underlying neuromodulation-induced cognitive enhancement in stroke patients across the microscale (single neurons), mesoscale (neuronal populations), and macroscale (interregional interactions). DLPFC: dorsolateral prefrontal cortex, M1: primary motor cortex, PPC: posterior parietal cortex, STG: superior temporal gyrus, MRI, magnetic resonance imaging; EEG, electroencephalography; fNIRS, functional near-infrared spectroscopy; MEG, magnetoencephalography. Figure created using BioGDP.com (7).

In this section, we reviewed neuromodulatory mechanisms at three scales: microscale (single neurons), mesoscale (neuronal populations), and macroscale (interregional interactions). Indeed, a comprehensive understanding of neuromodulatory mechanisms cannot be attained by examining any individual scale in isolation. From the perspective of systems neuroscience, stroke recovery is a complex, multiscale process emerging from the interplay among microscopic, mesoscopic, and macroscopic dynamics. To address this limitation, Shirinpour et al. (30) developed Neuron Modeling for TMS (NeMo-TMS), a multiscale modeling toolbox enabling simultaneous investigation of single-neuron responses to TMS across macro-, meso-, micro-, and subcellular scales. By integrating experimental findings into a unified computational framework, NeMo-TMS allows researchers to probe TMS mechanisms, thereby facilitating cross-scale understanding. Currently, this advanced technology has not yet been directly applied to stroke research. Further developments can be envisioned to use NeMo-TMS to validate the accuracy of neuronal membrane voltage and calcium dynamics in the neuromodulation of PSCI patients, thus facilitating a deeper understanding of PSCI’s physiological mechanisms and guiding the optimization of treatment parameters (**Figure 2A**).

## 4 Applications of brain function detection in neuromodulation

Neuroimaging primarily captures whole-brain neural activity and characterizes the spatiotemporal dynamics of brain networks. A multimodal magnetic resonance imaging (MRI) approach, coupling diffusion tensor imaging (DTI)-based structural connectivity (SC) and resting-state functional MRI (rs-fMRI)-based FC, offers a more comprehensive understanding of the neurological characteristics and intrinsic correlation properties in stroke patients (31). Cortical areas in PSCI patients exhibited lower FC-SC coupling in the occipital and frontal lobes compared to healthy individuals and stroke patients without cognitive impairment. Additionally, FC-SC coupling strength in the precuneus, paracentral lobule, and precentral gyrus correlates positively with cognitive performance. Three months after the stroke, increased FC–SC coupling strength was observed in the precentral gyrus and paracentral lobule, potentially reflecting an adaptive mechanism during cognitive recovery (32). fNIRS research indicates that PSCI patients show significantly reduced interhemispheric and intra-right hemispheric FC in comparison to healthy individuals, particularly in the somatosensory cortex, dorsolateral PFC, and medial PFC (33). Magnetoencephalography (MEG) studies provide evidence that early post-stroke cognitive dysfunction has a network-level basis. During early recovery, interhemispheric connectivity from the ipsilesional to the contralesional cortex increases (34). Finally, using machine learning models to analyze electroencephalography (EEG)-derived brain networks also help elucidate the neural mechanisms underlying stroke and recovery (35).

The integration of various brain function detection has gained popularity in neuroscience, driven by the understanding that no single method can comprehensively reveal neural activity complexity. For example, integrating multiple macroscale neuroimaging modalities provides valuable insights into the anatomical substrate and pathophysiological mechanisms, contributing to predictive models of outcomes (36). The EEG-MRI fusion technique can reveal brain network connectivity, neurovascular coupling, and neural oscillations across both temporal and spatial dimensions, thereby enhancing the understanding of the dynamic neuromechanisms in the progression of PSCI (37). The EEG-fNIRS fusion technique can also evaluate neurovascular coupling mechanisms. However, compared to the EEG-MRI fusion technique, it can better synchronize the rapid, dynamic changes in brain activity during complex tasks and dynamic environments (38). The MRI-fNIRS fusion technique combines the accurate spatial mapping of MRI with the ability of fNIRS to rapidly capture hemodynamic fluctuations, allowing researchers to associate cortical activity with specific brain regions in real time (39). Future research should consider using AI to integrate multimodal neuroimaging. Moreover, integrating brain function detection across various scales, such as combining optogenetics, chemogenetics, Neuropixels probes, two-photon microscopy with neuroimaging, may provide biomarkers relevant to PSCI and help guide stimulation dose selection and target optimization in humans, thereby strengthening the translation from basic research to clinical practice (**Figure 2B**).

## 5 Neuromodulation techniques for post-stroke cognitive impairment

### 5.1 Transcranial magnetic stimulation

To illustrate the efficacy of rTMS, we referred to a network meta-analysis with a large sample size that focused specifically on PSCI. This study evaluated 74 studies (n = 5478). The majority of these studies were randomized controlled trials (RCTs) with sham controls, although most involved small pilot samples (n < 50). The extant evidence suggests that rTMS can improve cognitive and daily life abilities of PSCI patients, with most studies targeting the DLPFC (40). DLPFC stimulation can enhance the deactivation of default mode network (DMN) nodes and facilitate cognitive processing under high demand, making it a preferred target for cognitive recovery (41). HF-rTMS and iTBS targeting the left DLPFC enhanced global cognition by increasing corticospinal excitability and instantly inducing theta oscillations, respectively, with HF-rTMS exerting a greater modulatory effect on attentional function than iTBS (28, 42, 43). LF-rTMS primarily targets DLPFC in the unaffected hemisphere to enhance cognitive function, and it reduces the number of magnetic stimulation pulses during treatment, thereby improving safety and patient tolerability (44). Mechanistically, LF-rTMS inhibits excitability in the contralateral cortex, thereby reducing its suppressive effect on the injured hemisphere and contributing to the restoration of interhemispheric inhibitory balance (45). The interhemispheric competition model may predominate in stroke patients with high structural reserve (the preservation of neural pathways and connectivity), whereas recovery in those with low structural reserve may be better explained by a hemispheric compensatory model (46). The hemispheric compensation model posits that when damage to the injured hemisphere is extensive and structural reserve is limited, rTMS excitation of the injured hemisphere is insufficient to support functional recovery. Instead, enhancing excitability in the healthy hemispheres is necessary to optimize treatment outcomes by expanding its compensatory functional capacity (47). Although the left DLPFC is the most commonly targeted region for TMS treatment of PSCI, the aforementioned bimodal balance-recovery model suggests that stimulation protocols should be adapted to the specific needs of individual patients. Furthermore, research has demonstrated that LF-rTMS targeting the contralesional primary motor cortex (M1) can enhance global cognitive function and visuospatial memory recall in stroke patients (48, 49). TMS targeting the posterior parietal cortex (PPC) has demonstrated therapeutic efficacy in stroke patients with unilateral spatial neglect (50). Using neuro-navigation to correct any positional or angulation errors of the TMS coil during the session will improve the accuracy of the target area (51).

In addition to the stimulation site, rTMS involves several key parameters, including frequency, intensity, and the number of pulses (52). In clinical settings, LF-rTMS is typically administered at 0.5 Hz or 1 Hz to reduce neuronal activity and cortical excitability, whereas HF-rTMS generally employs frequencies of 3 Hz, 5 Hz, 10 Hz, or 20 Hz to increase neuronal activity and cortical excitability. iTBS, a specific form of rTMS that replicates the brain’s innate theta rhythm, consists of delivering 3 pulses of 50 Hz bursts at 5 Hz (2s on and 8s off) for a total of 600 pulses, with each session lasting about 3 min (53). Using iTBS can instantly induce theta oscillations, which play a critical role in cognitive function and memory formation (43). Stimulus intensity varied between 70% and 120% of the resting motor threshold (RMT), with most studies selecting 80% RMT. The number of pulses varies significantly across studies (52). Different TMS parameters may influence outcomes in PSCI. However, these effects are not discussed in detail here, as they have been systematically examined in previous meta-analyses. In summary, a major challenge in the clinical practice of rTMS is the wide variation in the combination of parameters, and conducting orthogonally designed RCTs could help address this issue (**Table 1**).

**Table 1:** Application of TMS on PSCI patients in preclinical and clinical studies in the last 3 years.

Study	Type of stroke	Stroke stage	Age	Stimulation site	Stimulation parameter	Course of treatment	Evaluation tools	Main findings	Adverse events
Preclinical studies									
Chen 2023 (54)	MCAO rats	Subacute	7 weeks	Left frontal parietal lobe	The stimulus intensity was set to 33% of the maximum stimulator output. Stimulation was delivered at 10 or 20 Hz for 5 s, followed by a 60-s rest, repeated 10 times (totaling 500 or 1000 pulses per day).	Once daily for 3 weeks.	mNSS, MWM, LFB staining, ELISA, western blot, IF staining	rTMS facilitated M2 microglial polarization, reduced neuroinflammation and white matter lesions, thereby improving cognitive function. 20 Hz rTMS exhibited superior efficacy compared to 10 Hz rTMS.	Not reported
Hong 2024 (11)	tMCAO rats	Chronic	7 weeks	Left frontal parietal lobe	Stimulation was delivered at 20 Hz for 10 s with a 50 s rest period, for a total of 10 cycles.	Once daily for 3 weeks.	Shuttle box test, MWM, TEM, western blot	rTMS ameliorated cognitive impairment by regulating synaptic plasticity and increasing PSD95, SYN, and BDNF expression in the hippocampus.	Not reported
Jiang 2025 (55)	MCAO rats	Subacute	Adult	5 mm to the right of the bregma	The stimulus intensity was set to 120% RMT. Stimulation was delivered at 10 Hz for 3 s with a 50 s rest period, totaling 300 pulses.	Once daily for 7 consecutive days.	mNSS, MWM, rotarod test, IF staining, FJB staining, IHC staining, HE staining, TEM	rTMS attenuated hippocampal cell apoptosis and promoted axonal and myelin regeneration in the hippocampus, thereby enhancing spatial learning and memory.	
Luo 2025 (56)	MCAO rats	Acute	7 weeks	5 mm to the right of the bregma	The stimulus intensity was set to 120% RMT. Stimulation was delivered at 10 Hz for 3 s with a 50 s rest period, totaling 300 pulses.	Once daily for 7 or 14 consecutive days.	MRI, Grip strength, NORT, western blot	rTMS mitigated myelin sheath loss and promoted white matter repair, thereby enhancing motor and cognitive function.	Not reported
Luo 2023 (57)	MCAO rats	Acute	8–10 weeks	Cover the head	Stimulation was delivered at 10 Hz in 60 trains, each consisting of 20 pulses, with a 10 s inter-train interval, totaling 1200 pulses.	Once daily for 3, 7, 14, or 28 days within a 2-day period each week.	MRI, MWM, NORT, IF staining, RNA sequencing, western blot, Real-time PCR	rTMS promoted neural stem cell proliferation in the ischemic penumbra and improved cognitive function.	Not reported
Clinical studies									
Li 2025 (58)	Isch/hem	Acute	62.9 ± 9.36	DLPFC	The stimulus intensity was set to 80% RMT. Stimulation was delivered at 10 Hz with a 4 s interval for 20 sequences.	20 min/session, once daily for 4 weeks.	Visual n-back test, MoCA, ELISA	After rTMS treatment, a positive correlation was observed between increased serum levels of BDNF, NGF, 5-HT, and 5-HIAA and cognitive improvement.	Not reported
Liu 2024 (59)	Isch/hem	Subacute and chronic	56.04 ± 8.00	Left DLPFC	The stimulus intensity was set to 90% RMT. Stimulation was delivered at 10 Hz for a total of 1,280 pulses.	18 min/session, once daily for 2 weeks.	fNIRS, visual n-back test, MoCA, MMSE, DST, WCST, SDMT	rTMS improved working memory, attention, and executive function, possibly as a result of the neural activation in the left DLPFC, right premotor cortex, and right superior parietal lobule.	Headache, infections, eyelid twitching, and dizziness
Ren 2025 (60)	Isch/hem	Subacute and chronic	58.57 ± 7.89/ 60.77 ± 8.05	Left personalized frontoparietal cognitive network	The stimulus intensity was set to 80% RMT. Each iTBS cycle consisted of a 10-s period: 2 s of three 50-Hz bursts at 5 Hz, followed by an 8-s rest. Patients in the high-dose group underwent two 600-s sessions (3600 pulses/day), whereas those in the low-dose group underwent two 200-s sessions (1200 pulses/day).	Twice daily for 15 consecutive days.	MoCA, WMS-RC, WAIS-RC, MMSE	iTBS enhanced working memory and attention in patients, exhibiting both high-dose efficacy and dose-dependent effects.	Scalp pain, drowsiness, nausea, facial twitching, dizziness, and fatigue
Jiang 2025 (61)	Isch/hem	Acute and subacute	52.97 ± 8.79/53.50 ± 8.83	Left DLPFC/Right DLPFC	The stimulus intensity was set to 100% RMT. Stimulation was delivered at 20 Hz in 50 trains of 20 pulses each, separated by 30-s intertrain intervals.	20 min/session, once daily for 20 consecutive days.	MoCA, LOTCA, ERP P300, modified Barthel index	rTMS targeting the left DLPFC improved global cognition, language, and orientation, while rTMS targeting the right DLPFC improved visual and spatial perception.	Not reported
Zhang 2023 (62)	Isch/hem	Subacute	57.20 ± 6.41	Cerebellum	The stimulus intensity was set to 80% of the active motor threshold. Stimulation was delivered as bursts of 3 pulses at 50 Hz, repeated at 5 Hz (2 s on and 8 s off) for a total of 600 pulses.	4 to 5 min/session, 5 times per week for 2 weeks.	EEG, HMDS, HAMA, MADRS, IDSSR, MMSE, MoCA, WMT, BNT	iTBS effectively regulated the cerebello-limbic-parietal circuit and alter microstate dynamics related to these regions, thereby enhancing emotional and cognitive functions.	Not reported

MCAO, Middle Cerebral Artery Occlusion; mNSS, Modified Neurological Deficit Score; MWM, Morris Water Maze; ELISA, Enzyme-Linked Immunosorbent Assay; LFB staining, Luxol Fast Blue Staining; IF staining, Immunofluorescence Staining; FJB staining, Fluoro-Jade B Staining; IHC staining, Immunohistochemistry Staining; HE staining, Hematoxylin-Eosin Staining; TEM, Transmission Electron Microscopy; tMCAO, Transient Middle Cerebral Artery Occlusion; SYN, Synaptophysin; PSD-95, Postsynaptic Density Protein 95; NORT, Novel Object Recognition Test; MRI, Magnetic Resonance Imaging; Isch, Ischemic; Hem, Hemorrhagic; DLPFC, Dorsolateral Prefrontal Cortex; RMT, Resting Motor Threshold; MoCA, Montreal Cognitive Assessment; BDNF, Brain-Derived Neurotrophic Factor; NGF, Nerve Growth Factor; fNIRS, Functional Near-Infrared Spectroscopy; MMSE, Mini-Mental State Examination Score; DST, Digit Span Test; WCST, Wisconsin Card Sorting Test; SDMT, Symbol Digit Modalities Test; WMS-RC, Wechsler Memory Scale-Revised in China; WAIS-RC, Chinese Revision of the Wechsler Adult Intelligence Scale; LOTCA, Loewenstein Occupational Therapy Cognitive Assessment; ERP, Event-Related Potential; EEG, Electroencephalography; HMDS, Hamilton Depression Scale; HAMA, Hamilton Anxiety Scale; MADRS, Montgomery-Asberg Depression Rating Scale; IDSSR, Depressive Symptom Self-Rating Scale; WMT, Working Memory Test; BNT, Boston Naming Test.

### 5.2 Transcranial electrical stimulation

TES administers a low-intensity electric current (1–4 mA) to the scalp, typically via two or more electrodes. According to the type of the applied current, TES can be categorized into tDCS, tACS, tRNS, transcranial pulsed current stimulation (tPCS), and temporal interference stimulation (TI) (63). The small size and portability of tDCS enhance its value as a neuromodulation tool (64). A clinical trial demonstrated that home-based tDCS with remote supervision can facilitate cognitive recovery in chronic stroke patients (65). When targeting deeper brain regions, such as the hippocampus (a region central to memory-related tasks) or the anterior cingulate cortex (a region involved in interference control), tDCS lacks sufficient penetration and focality to precisely target these areas without affecting the overlying cortex (66). However, the recently developed HD-tDCS can deliver a more focused current to the target region, partially addressing this limitation (67). Unlike TMS, tDCS does not directly trigger action potentials but modulates the resting membrane potential by regulating sodium-calcium channels and NMDARs, thereby altering the probability of neuronal discharge (68).

The mechanism of tACS differs fundamentally from that of tDCS in that no effect is observed on the average membrane potential. During each cycle, one electrode serves as the anode and the other as the cathode. In the subsequent cycle, the roles of the electrodes are reversed, ensuring that the average membrane potential remains unaltered (66). tACS targeting the supplementary motor area (SMA) could improve speech comprehension in stroke patients (69). Furthermore, a recent study developed cross-frequency (alpha-gamma) bifocal tACS (cf-tACS) by applying low-power sinusoidal currents at distinct frequencies to the primary visual cortex and the medio-temporal area. This study provides first evidence that a single session of cf-tACS can modify inter-areal coupling in healthy and stroke participants; however, long-term treatment is needed to further verify the behavioral effects (70). tRNS is a variant of tACS that transmits alternating currents at random intensities. In most tRNS studies, a frequency spectrum ranging from 0.1 Hz to 640 Hz (full spectrum) or from 101 Hz to 640 Hz (high-frequency stimulation) was employed (71, 72). It was proposed that tRNS enhances neural firing synchronization by amplifying subthreshold oscillatory activity, thereby reducing endogenous noise. This higher signal-to-noise ratio within the central nervous system, along with increased sensitivity in sensory processing, may contribute to augmented perception and cognitive performance (73). Of note, tRNS has not yet demonstrated therapeutic benefits for PSCI (**Table 2**).

**Table 2:** Application of TES on PSCI patients in preclinical and clinical studies in the last 5 years.

Study	Type of stroke	Stroke stage	Age	Stimulation site	Stimulation parameter	Course of treatment	Evaluation tools	Main findings	Adverse events
tDCS									
Ai 2024 (74)	Isch/hem	Subacute	55.25± 6.27	Left DLPFC	Stimulation intensity was set to 2 mA.	30 min/session, 5 times a week for 2 weeks.	EEG, fNIRS, MoCA, BDST, gene polymorphism detection	tDCS enhanced cognitive function by increasing left frontoparietal connectivity and attenuating the rise in slow oscillatory activity. Methionine carriers exhibited greater cognitive improvements and heightened sensitivity to tDCS.	Two participants dropped out of the study due to adverse effects.
Jung 2024 (75)	MOCA mice	Subacute	6 weeks	Motor cortex	High-definition-tDCS was applied at a current density of 43 µA/mm² and a charge density of 51.6 kC/m².	20 min/session, once daily for 7 consecutive days.	Corner test, Pole test, OFT, NOR, trace fear conditioning, HE staining, Nissl staining, RNA sequencing, Real-time PCR, enzyme-linked immunosorbent assay, western blot	High-definition-tDCS demonstrated superior efficacy compared to conventional tDCS. It mitigated neuronal death and inflammation in the peri-infarct region through NMDA receptor–dependent SREBP1 signaling, thereby enhancing motor and cognitive recovery.	Not reported
Kaviannejad 2021 (76)	CI rats	Acute	Adult	Between Lambda and Bregma points	Stimulation intensity was set to 400 μA.	15 min/session for once.	ELISA, HE staining, TUNEL staining, oxidative stress assay, western blot, IF staining	tDCS decreased neuronal death and apoptosis in the hippocampal CA1 region.	Not reported
Zanão 2024 (77)	Isch/hem	Subacute and chronic	62.2 ± 12.3	DLPFC	Stimulation intensity was set to 2 mA.	30 min/session, 5 times a week for 2 weeks, then once per week.	MoCA, MMSE, FAB, trail making test, WAIS-III, stroop color and word test	No consistent cognitive improvements were observed following tDCS, and its effects may vary by the severity of depression and the complexity of tasks.	Not reported
Zheng 2025 (78)	Isch/hem	Chronic	63.4 ± 12.7	Right cerebellar cortex	Stimulation intensity was set to 2 mA.	20 min/session, once daily for 5 consecutive days.	COAST, Carer COAST, SAQOL-39, PROMIS-Global	tDCS did not lead to significant improvements in language outcomes but enhanced certain aspects of quality of life.	No severe adverse events
tACS									
Bevilacqua (70) 2024	Isch/hem	Subacute and chronic	24.6 ± 11.2	V1 and MT on the lesioned hemisphere	Stimulation frequency was set to the individual peak frequencies of α (8–12 Hz) and γ (30–45 Hz). Stimulation intensity was set to 3 mA.	25 min/session for once.	EEG, CDDI	One session of cross-frequency bifocal tACS modified inter-areal coupling but was insufficient to produce lasting behavioral effects.	Not reported
Schuhmann (24) 2022	Isch/hem	Subacute	57.8 ± 9.7	Contralesional PPC	Stimulation frequency and intensity were set to 10 Hz and 1.5 mA.	Maximum 30 min per session, twice on separate days with ≥1-day interval.	CVDT, BT, LBT	tACS at alpha frequency enhanced spatial attention by supporting the activity of unilateral alpha oscillations.	Not reported
Spanje 2024 (79)	Isch/hem	Chronic	61.00 ± 20.50	Contralesional PPC	Stimulation frequency and intensity were set to 10 Hz and 1.5 mA.	Maximum 40 min per session, 3 times a week for 3 weeks.	SCT, CVDT, MLBT-d, SLBT, BTT, CBS, SNQ	tACS at alpha frequency improved visual search performance and perception on the neglected side, with effects lasting for 3 months.	Not reported
Xie 2022 (69)	Isch/hem	Chronic	Mean age = 59 years	SMA	Stimulation frequency and intensity were set to 6 Hz and below 2 mA	30 min/session, once daily for 14 consecutive days.	ABC	tACS enhanced the effectiveness of speech and language therapy, particularly in improving speech comprehension.	Not reported
tRNS									
Gong 2024 (80)	VCI	Not reported	60–80 years	left inferior parietal lobe	Stimulation will be delivered at a frequency range of 101–640 Hz and an intensity of 2 milliamps.	20 min/session, once daily for 2 weeks.	MRI, DTI, PSQI, ANT, CVFT, HK MoCA	Not applicable	Not applicable

Isch, Ischemic; Hem, Hemorrhagic; EEG, Electroencephalography; fNIRS, Functional near-infrared spectroscopy; MoCA, Montreal Cognitive Assessment; BDST, Backward Digit Span Test; CI, Cerebral Ischemia; MoCA, Montreal Cognitive Assessment; OFT, Open-field Test; NOR, Novel Object Recognition; HE staining, Hematoxylin and Eosin Staining; IF staining, Immunofluorescence Staining; NMDA, N-Methyl-D-Aspartate; SREBP1, Sterol Regulatory Element-Binding Protein 1; ELISA, Enzyme-Linked Immunosorbent Assay; TUNEL staining, Terminal Deoxynucleotidyl Transferase dUTP Nick End Labeling Staining; MMSE, Mini-Mental State Examination Score; FAB, Frontal Assessment Battery; WAIS-III, Wechsler Adult Intelligence Scale; WAB-R, Western Aphasia Battery-Revised; COAST, Communication Outcomes After Stroke; SAQOL-39, Stroke and Aphasia Quality of Life Scale-39; PROMIS-Global, Patient-Reported Outcomes Measurement Information System; V1, Primary Visual Cortex; MT, Medio-Temporal Area; CDDI, Coarse Direction Discrimination and Integration; PPC, posterior parietal cortex; CVDT, Computerized Visual Detection Task; BT, Bell’s Task; LBT, Line Bisection Task; SCT, Star Cancellation Task; MLBT-d, McIntosh Line Bisection Task-Digitized; SLBT, Schenkenberg Line Bisection Task; BTT, Baking Tray Task; CBS, Catherine Bergego Scale; SNQ, Subjective Neglect Questionnaire; SMA, Supplementary Motor Area; ABC, Aphasia Battery of Chinese; VCI, Vascular Cognitive Impairment; MRI, Magnetic Resonance Imaging; DTI, Diffusion Tensor Imaging; PSQI, Pittsburgh Sleep Quality Index; ANT, Attention Network Test; CVFT, Category Verbal Fluency Test; HK MoCA, Montreal Cognitive Assessment Hong Kong Version.

tPCS is a recent advance in neuromodulation that converts direct current into pulses with programmable parameters, including pulse duration and inter-pulse interval (81). The primary mechanisms include charge summation by pulsed currents, which generates larger voltage gradients across neuronal ensembles, and phasic, subthreshold modulation within the region of interest induced by pulsed current stimulation (82). It has been demonstrated that tPCS exerts a frequency-specific effect on electroencephalographic band power, regulates functional connectivity, promotes interhemispheric coherence, and facilitates cortical plasticity (83). A case report provided preliminary evidence that tPCS targeting the bilateral DLPFC, parietal regions, and the precuneus improved memory, recognition, and other cognitive functions in patients with PSCI (84). TI represents another noninvasive approach for achieving effective deep brain neuromodulation. TI employs two high-frequency alternating currents of slightly different frequencies, delivered through two pairs of scalp electrodes. This approach produces a low-frequency modulated electric field (envelope modulation), enabling stimulation of deep structures while minimizing direct neural activation in the superficial cortex(F. 85). By oscillating the modulation envelope, TI can synchronize with ongoing neural activity through neural entrainment, similar to tACS (86). Violante et al. (87) applied sinusoidal currents of 2005 Hz and 2000 Hz to generate a 5 Hz envelope modulation targeting the left hippocampus, thereby achieving selective modulation of neural activity in the intended region. This intervention improved recall accuracy, suggesting that TI has the potential to enhance cognitive performance. An RCT will compare the efficacy of TI targeting the hippocampal region with tACS targeting the DLPFC in patients with PSCI, using standardized cognitive assessments and fNIRS to probe the underlying neural mechanisms (ChiCTR2400081207) (88).

### 5.3 Vagus nerve stimulation

VNS involves invasive VNS (iVNS) and non-invasive VNS, also called transcutaneous VNS (tVNS). tVNS is further subdivided into auricular (taVNS) and cervical (tcVNS) subtypes (89). taVNS offers a safe and well-tolerated alternative, engaging vagal pathways and projections with physiological responses similar to iVNS (90). MRI has shown that VNS causes localized alterations of blood flow within brain structures associated with cognition, including brainstem, hypothalamus, thalamus, amygdala, and hippocampus (91). In preclinical stroke studies, VNS has been demonstrated to decrease infarct volume and neurological deficits, thereby enhancing cognitive function by activating the cholinergic anti-inflammatory pathway, modulating blood-brain barrier integrity, and promoting angiogenesis and neurogenesis (92). An 8-week study by Chen et al. (93) showed that taVNS induced white matter remodeling in the bilateral DLPFC and enhanced global cognitive and executive function in a patient with chronic cerebral infarction. Despite its preliminary therapeutic potential, VNS for PSCI remains largely confined to preclinical studies and early-stage clinical investigations. PSCI is a chronic and heterogeneous condition that often requires long-term intervention to achieve functionally meaningful benefits. Whether improvements on short-term neuropsychological tests translate into real-world functional gains requires confirmation in large-scale clinical trials with long-term follow-up. Furthermore, the clinical outcomes of VNS are closely related to stimulation parameters, including stimulation site, on/off cycles, waveform, current amplitude, and frequency. Therefore, it is essential to elucidate these parameters to optimize VNS efficacy (**Table 3**).

**Table 3:** Application of VNS on PSCI patients in preclinical and clinical studies in the last 10 years.

Study	Type of stroke	Stroke stage	Age	Stimulation site	Stimulation parameter	Course of treatment	Evaluation tools	Main findings	Adverse events
Preclinical studies									
Gong 2025 (94)	MCAO mice	Subacute	8–9 weeks	Left cervical vagus nerve	1 mA, 330 ms pulse width	30 min/session, once daily for 5 consecutive days.	IHC staining, western blot, oxidative stress assay, ICP‐MS, TTC, Mnss, grid walking test, pole test, Barnes Maze, TEM	TaVNS promoted neurological and cognitive recovery by inhibiting ferroptosis through activation of α7nAChR.	Not reported
Liu 2016 (17)	MCAO/R rats	Acute	Adult	Left cervical vagus nerve	1 mA, 20 Hz, 0.4 ms pulse width	10 min/session, once before and once after surgery.	WMT, automated shuttle box test, western blot	VNS improves spatial and fear memory, possibly through norepinephrine release.	Not reported
Clinical studies									
Colombo 2023 (29)	Isch/hem	Acute and chronic	61.9 ± 11.22	Left cervical vagus nerve	mean = 0.8 mA	Single session	Box and blocks test, indexing manual dexterity, Go/No-go task, MMSE, FAB, DSF, DSB	One session of tVNS did not appear to enhance cognitive function.	Not reported
Chen 2024 (93)	Isch	Chronic	71 years	Right cervical vagus nerve	a dilatational wave of 20/4 Hz	30 min/session, twice daily, 5 days a week for 8 weeks	DTI, MoCA, MMSE, AVLT, STT, HAMD, HAMA, PSQI	TaVNS influenced prefrontal-subcortical networks associated with executive functioning and emotional modulation.	No discomfort
Li 2022 (95)	Isch	Acute	45–75 years	Left cervical vagus nerve	4–6 mA, 5 Hz, 200 μs wave width	30 min/session, twice daily, 5 days a week for 8 weeks	MMSE, MoCA, MBI, SF-36, serum oxidative stress assay, safety evaluation	Not applicable	Not applicable

MCAO, Middle Cerebral Artery Occlusion; IHC staining, Immunohistochemistry Staining; ICP‐MS, Inductively Coupled Plasma Mass Spectrometry; TTC, Triphenyltetrazolium Chloride; mNSS, Modified Neurological Severity Score; TEM, Transmission Electron Microscope; α7nAChR, α7 Nicotinic Acetylcholine Receptor; MCAO/R, Middle Cerebral Artery Occlusion/Reperfusion; WMT, Working Memory Test; Isch, Ischemic; Hem, Hemorrhagic; MMSE, Mini-Mental State Examination Score; FAB, Frontal Assessment Battery; DSF, Digit Span Forward; DSB, Digit Span Backward; DTI, Diffusion Tensor Imaging; MoCA, Montreal Cognitive Assessment; AVLT, Auditory Verbal Learning Test-HuaShan Version; STT, Shape Trails Test; HAMD, Hamilton Depression Scale; HAMA, Hamilton Anxiety Scale; PSQI, Pittsburgh Sleep Quality Index; MBI, Modified Barthel Index; SF-36, 36-item Short-Form Health Survey.

### 5.4 Photobiomodulation

PBM is a non-invasive, non-thermal form of neuromodulation in which cells and tissues are exposed to photons within specific wavelength ranges (96). Lasers and light-emitting diodes (LEDs) are the two most commonly used light sources in PBM. The precisely defined wavelengths of lasers are essential for experiments involving photosensitive compounds or mechanistic investigations. The high directionality and energy density of lasers enable precise targeting of the intended region, making them optimal for applications necessitating accurate, deep stimulation in medical practice. In contrast, LEDs offer a broader spectral range, making them more appropriate for general illumination and large-scale biological neuromodulation (97). Gao et al. (98) have demonstrated that laser-based PBM is more effective than LED-based PBM in enhancing cognitive function in patients. This finding warrants careful consideration, given that LEDs in clinical applications are safer, offer ease of home use, enable integration into wearable devices, and are low cost (99). Continuous wave and pulsed wave represent two principal modes of light energy output. Continuous wave is more appropriate for conditions that require long-term and steady irradiation, such as pain management, soft tissue inflammation, and wound healing. Pulsed wave exhibits remarkable efficacy in applications necessitating high peak power and expeditious response times (97). Tang et al. (100) employed psychological experiments and EEG to demonstrate that pulsed wave PBM produced greater cognitive enhancement than continuous wave PBM and modulated high-frequency neural oscillations. The primary mechanism of PBM involves cytochrome c oxidase (CCO), the principal photoreceptor for red-to-NIR light in neuronal mitochondria (101). Photons absorbed by CCO displace inhibitory nitric oxide (NO), resulting in increased mitochondrial membrane potential, enhanced oxygen consumption, and greater ATP production, which collectively reduce oxidative stress and neuroinflammation, ultimately mitigating neuronal damage following stroke (102).

Preclinical evidence indicates that PBM inhibits NOD-like receptor family pyrin domain-containing 3 (NLRP3) inflammasome activation and interleukin-1beta (IL-1β) expression in astrocytes of the hippocampal CA1 region, thereby promoting neural stem cell differentiation into neurons and supporting cognitive recovery (103). A clinical trial in stroke patients reported that, following the administration of transcranial photobiomodulation (tPBM) in combination with neuromuscular electrical stimulation, the experimental group showed a greater increase in mean Mini-Mental State Examination (MMSE) scores (24.85 ± 1.67 vs. 28.00 ± 1.73; *p* < 0.05) than the control group (26.28 ± 2.13 vs. 26.42 ± 1.27; *p* > 0.05) (104). Studies on healthy individuals provided a potential explanation for this phenomenon. Research has shown that tPBM targeting the right PFC can enhance visual WM capacity and elevate occipitoparietal contralateral delay activity (105). Additionally, tPBM modulates brain network topology, synchronization, and complexity, thereby facilitating more efficient neural information processing (106). Notably, direct clinical evidence supporting PBM for the treatment of PSCI remains limited. Future studies that examine light irradiation via the nasal and oral cavities, or the application of light-controlled nanomaterial drug-delivery systems to reduce light attenuation by the skull while enhancing safety, may further extend the clinical applications of PBM (**Table 4**).

**Table 4:** Application of PBM on PSCI patients in preclinical and clinical studies in the last 10 years.

Study	Type of stroke	Stroke stage	Age	Stimulation site	Stimulation parameter	Course of treatment	Evaluation tools	Main findings	Adverse events
Preclinical studies									
Kim 2022 (107)	PT and MCAO mice	Acute	Not reported	Sensorimotor cortex of the ipsilesional region	LED (630 nm, 850 nm, and 940 nm), 17 mW/cm^2^	20 min/session, (1) twice a day before or after surgery for 3 days; (2) immediately after surgery, once daily for 7 days	TTC, infarct volume, neurological scores, wire-grip test, elevated plus maze test, MWM, IF staining, western blot	PBM enhanced spatial learning and memory by attenuating pyroptosis and regulating microglial polarization within the hippocampus and cortex, with the 630-nm PBM demonstrating the greatest efficacy.	Not reported
Yang 2025 (108)	tMCAO rats	Subacute	Not reported	5 cm above the right parietal bone	LED (638 nm, 755 nm, and 808 nm), 20 mW/cm^2^	10 min/session, once daily for 7 consecutive days.	mNSS, OFT, MWM, NOR, MRI, ELISA, IF staining,	Using 755-nm PBM enhanced molecular transport in the extracellular space of the brain and reduced cognitive impairment.	Not reported
Yu 2017 (109)	MCAO rats	Subacute	8 weeks	Baekhoe and Sinmun	Laser acupuncture, 650 nm, 30 mW, 100 Hz	once every 2 days, for 2 weeks	MWM, acquisition trial, probe trial, IHC staining, real-time PCR,	Laser acupuncture promoted cognitive recovery by enhancing the cholinergic system in the hippocampal CA1 region and provided neuroprotective effects through modulating BDNF, CREB, Bax, and Bcl-2 gene expression.	Not reported
Clinical studies									
Estrada-Rojas 2023 (110)	Isch	Subacute	38 years	Left side of the scalp, primarily targeting the Sylvian fissure language areas, followed by stimulation of the whole scalp.	(1) LED cluster (630 nm, 660 nm, and 850 nm), 200 mW/cm^2^, 12 J/cm^2^ per min; (2) LED helmet (810 nm), 24 mW/cm^2^, 28.8 J/cm^2^	45 min/session, twice a week, for 30 sessions over 5 months	Clinical dysarthria evaluation, BDAE, Burns Left Hemisphere Inventory of Communication and Cognition	tPBM combined with speech-language therapy enhanced dysarthria and expressive language function.	Not reported
Naeser 2020 (111)	Isch/hem	Chronic	46–49 years	(1) Bilateral and midline placements, including SMAs at vertex; (2) left hemisphere (ipsilesional); (3) left hemisphere + one midline cortical node of DMN; (4) left hemisphere + two midline cortical nodes of DMN	LED (633 nm and 870 nm), 22.2mW/cm^2^	3 times/week, for 6 weeks	rs-fcMRI, BDAE, BNT, PNT	Optimal placement over the ipsilesional hemisphere combined with two midline DMN nodes was associated with improved naming ability.	No discomfort

PT, Photothrombosis; MCAO, Middle Cerebral Artery Occlusion; LED, Light-Emitting Diodes; TTC, Triphenyltetrazolium Chloride; MWM, Morris Water Maze; IF staining, Immunofluorescence Staining; tMCAO, Transient Middle Cerebral Artery Occlusion; mNSS, Modified Neurological Deficit Score; OFT, Open-Field Test; NOR, Novel Object Recognition; MRI, Magnetic Resonance Imaging; IHC staining, Immunohistochemistry Staining; CREB, cAMP Response Element-Binding Protein; BDNF, Brain-Derived Neurotrophic Factor; Bcl-2, B-Cell Lymphoma 2; Bax, Bcl-2-Associated X Protein; Isch, Ischemic; BDAE, Boston Diagnostic Aphasia Examination-Third Edition; Hem, Hemorrhagic; SMAs, Supplementary Motor Areas; DMN, Default Mode Network; rs-fcMRI, Resting-State Functional-Connectivity Magnetic Resonance Imaging; BNT, Boston Naming Test; PNT, Philadelphia Naming Test.

### 5.5 Transcranial ultrasound stimulation

TUS enables noninvasive, precise, stable, and targeted modulation of brain regions, making it an attractive option for both basic and clinical researchers (112). High-intensity focused ultrasound (HIFU) (I_SPPA_ > 200 W/cm^2^) is widely used for tumor thermocoagulation, intracerebral thrombus lysis, and the treatment of tremor associated with Parkinson’s disease and essential tremor. Low-intensity transcranial focused ultrasound (LIFU) (I_SPPA_ < 100 W/cm^2^) is regarded as a primary modality for non-invasive neuromodulation (113). This is because HIFU primarily relies on thermal effects, whereas LIFU mainly depends on mechanical bioeffects (114). Studies have shown that TUS can penetrate 5–6 cm into the cranium, enabling precise stimulation of deep brain lesions with millimeter-scale spatial resolution (115). To some extent, TUS overcomes the limitation that focal DBS currently requires the implantation of deep electrodes. Moreover, clinical neuromodulation requires the stimulation of pathological brain tissue, which often involves significant alterations in the brain’s normal conductivity. For electromagnetic techniques, accurately modeling these conductivity changes to achieve precise, individualized targeting remains nearly impossible. Conversely, ultrasound targeting is unaffected by such conductivity variations (116).

Multiple preclinical studies have indicated that TUS can restore cerebral blood supply and enhance the rate of vascular recanalization, attenuate inflammatory responses and apoptosis, and enhance neurotrophic factor expression, thereby conferring neuroprotection after stroke. However, whether TUS improves cognitive outcomes in stroke models remains largely unexplored (117–119). Only one clinical trial was identified that examined the effects of TUS in treating PSCI. Wang et al. (120) administered 20-minute daily sessions of TUS to the foreheads of stroke patients for six weeks. They reported that, compared to the sham stimulation group, patients in the TUS group exhibited significant improvements in executive function, naming, attention, language, and delayed recall, as well as higher levels of BDNF. Despite the positive results, the heterogeneity of testing conditions complicates the evaluation of these findings. Future research should test various parameter combinations and apply detailed cognitive function assessment tools to evaluate the effects of TUS for clinical practice.

### 5.6 Brain-computer interface

BCIs are emerging communication and control systems that could revolutionize medicine by connecting the brain to external devices (121). A recent review indicated that BCI not only detects brain activity but also provides a means for assessing and training cognitive function in PSCI patients, facilitates intention expression, addresses cognitive and memory deficits, and enhances communication (122). Neurofeedback training (NFT) and motor imagery (MI) training are widely used approaches for enhancing cognitive function in BCI applications. T et al. (123)designed an EEG-BCI-based NFT game and evaluated its effectiveness in enhancing attention and memory among stroke patients, using both behavioral assessments and neurophysiological indices. The results showed that EEG-based attention scores in stroke patients improved by 4.29% to 32.18% from the first to the third session. Considering the interaction between sensorimotor and cognitive systems, a study examined the effects of MI-BCI training on upper limb function and attention in this population. The study found that MI-BCI can detect brain activity, classify and extract relevant information, decode motor intentions, and facilitate interneuronal communication. These mechanisms contribute to the rehabilitation of upper limb function and attention (124). Future research should balance the strengths and weaknesses of invasive and non-invasive BCI approaches to identify a more effective method for signal acquisition, while integrating AI, machine learning, and neural networks to develop bidirectional and high-performance BCIs (**Table 5**).

**Table 5:** Application of BCI on PSCI patients in clinical studies in the last 5 years.

Study	Type of stroke	Stroke stage	Age	BCI facility	Course of treatment	Evaluation tools	Main findings	Adverse events
Endzelytė 2025 (125)	Isch/hem	Subacute	61.47 ± 12.65/ 63.05 ± 11.64	MI-BCI, BCI-FES	52 min/session, 2–3 times a week for a total 15 sessions	MMSE, CDT, FIM, BBT, 9HPT	Integration BCI into occupational therapy improved cognitive performance, motor function, and daily life independence.	Not reported
Wan 2025 (126)	Isch/hem	Acute/subacute	56.6 ± 11.60	MI-BCI, BCI-VR-PT system	30 min/session, 5 days a week for 4 weeks	MoCA, SDMT, TMT, average attention index, FMA-LE, TUG, BBS	The BCI-VR-PT system improved attention and lower-extremity function.	No discomfort
Musso 2022 (127)	Isch	Chronic	58 ± 11	BCI-based language training	30 h, 4 days per week	rs-fMRI, AAT, S&V, CAL, TAP	The BCI-based language training resulted in moderate to substantial generalized improvements in language function, irrespective of initial aphasia severity.	Not reported
Yuan 2021 (128)	Isch	Subacute	64.5 (58.2-67.5)	BCI-VR-PT system	6 times a week for 2 weeks	MMSE, MoCA, DST, SDMT, FMA-LE, attention index	The BCI-VR-PT system enhanced lower extremity motor function by facilitating patient engagement. Although improvements in attention scales were observed, these did not achieve statistical significance.	Not reported
Zhao 2022 (129)	Isch	Subacute	52.4 ± 11.2	BCI-robotic	30 min/session, 6 times a week for 4 weeks	MEP, serum BDNF levels LOTCA, FMA-LE, FAC, FMA-B, MBI	BCI-controlled robot training enhanced cognitive recovery and improved lower-extremity function.	Two participants dropped out of the study because of consent or dizziness.

Isch, Ischemic; Hem, Hemorrhagic; MI, Motor Imagery; FES, Functional Electrical Stimulation; MMSE, Mini-Mental State Examination Score; CDT, Clock Drawing Test; FIM, Functional Independence Measure; BBT, Box and Blocks Test; 9HPT, Nine-Hole Peg Test; VR, Virtual Reality; PT, Pedaling Training; MoCA, Montreal Cognitive Assessment; SDMT, Symbol Digit Modalities Test; TMT, Trail Making Test; FMA-LE, Fugl-Meyer Assessment for Lower Extremity; TUG, Timed “Up & Go”; BBS, Berg Balance Scale; rs-fMRI, Resting-State Functional Magnetic Resonance Imaging; AAT, Aachen Aphasia Test; S&V, Snodgrass & Vanderwart Naming Test; CAL, Communicative Activity Log; TAP, Test of Attentional Performance; DST, Digit Span Test; MEP, Motor-Evoked Potentials; BDNF, Brain-Derived Neurotrophic Factor; LOTCA, Loewenstein Occupational Therapy Cognitive Assessment; FAC, Functional Ambulation Category; FMA-B, Fugl-Meyer Assessment for Balance; MBI, Modified Barthel Index.

### 5.7 Other types of neuromodulation strategies

It has been hypothesized that TSS may enhance motor-related language functions, including action verb processing and speech articulation, by modulating activity within the sensorimotor network (130). Three studies performed by the same research group have evaluated the efficacy of TSS in patients with chronic post-stroke aphasia. They reported that anodal TSS improved verb naming performance, which was positively associated with cerebellar-cortical network connectivity. Additionally, patients showed greater accuracy in repeating the treated items (131–133).

Recent research suggests that DBS may rescue spatial memory in rats with cerebral ischemia by enhancing hippocampal synaptic activity (134). Although preclinical studies have indicated the potential efficacy of DBS in PSCI recovery, there is limited clinical evidence to support its use.

A meta-analysis involving 5,543 participants confirmed the therapeutic efficacy of MST in treating PSCI (135). Research indicated that MST can enhance depression, cognitive function, daily living activities, and neurological outcomes in stroke patients. The therapeutic mechanisms of MST may involve the evocation of positive emotions and relaxation, the influence of the autonomic and neuroendocrine systems, and the activation of the frontal, parietal, cerebellar, and limbic areas. Due to its susceptibility to personal preferences, emotional status, and cultural differences, MST may be better suited as an adjunct to other neuromodulation strategies (136).

### 5.8 Gene-based neuromodulation

Compared to traditional neuromodulation, genetic-based neuromodulation can simultaneously regulate multiple brain regions and produce more sustained effects (137). A recent study demonstrated that chemogenetic activation of neurons in the dorsal dentate gyrus alleviated anxiety, and depression in infarcted mice (138). Similarly, chemogenetic inhibition of ventral hippocampal CA1 pyramidal neurons reduced neurological injuries, anxiety, depression, and pain perception induced by cerebral ischemia through the cAMP response element-binding protein (CREB)-BDNF pathway (139). Furthermore, Chen et al. (140) found that early-stage chemogenetic activation of parvalbumin neurons ameliorated spatial working memory deficits induced by prefrontal ischemia in the T-maze task. Further studies revealed that parvalbumin neurons were suppressed during global ischemia but quickly recovered upon reperfusion. However, optogenetic stimulation of parvalbumin neurons induced GABAergic synaptic network activity remained significantly suppressed even one hour after reperfusion, potentially contributing to sensory and cognitive impairments observed after transient global ischemia (141).

Although chemogenetics and optogenetics have been demonstrated to precisely regulate neuronal excitability within specific brain regions, most studies have focused primarily on post-stroke motor dysfunction, while their direct application in models of PSCI remains relatively limited. Future research should focus on ensuring efficient receptor delivery and stable expression, improving ligand specificity, identifying the optimal timing and duration of genetic-based neuromodulation strategies, and clarifying the long-term consequences of chronic neural circuit modulation. Furthermore, emerging genetic-based neuromodulation strategies based on sound or magnetic stimulation may be more acceptable to patients due to their non-invasive or minimally invasive nature.

## 6 Multi-target neuromodulation for post-stroke cognitive impairment: from cognitive enhancement to motor function recovery

A fNIRS study reported that patients with stroke exhibit asymmetric changes in bilateral hemispheric FC, which may result in increased hemispheric response independence. Specifically, FC is significantly lower in the right hemisphere of PSCI patients than in the left, and the right central executive network shows a strong association with cognitive function (142). Given the imbalance between the bilateral hemispheres in PSCI patients, the target of neuromodulation may shift from a single area to multiple areas or the entire brain network, thereby enhancing the functional activity of the entire cognitive network.

Human cognition is primarily supported by several key brain networks, including the DMN, executive control network (ECN), salience network (SN), dorsal attention network (DAN), and frontoparietal network (FPN) (143). Notably, the core regions of both ECN and FPN include DLPFC. DLPFC serves as a key target within the frontal cortex-subcortical circuitry and plays a critical role in regulating cognition, memory, attention, and executive function (44). DLPFC plays a crucial role in cognitive-motor tasks under conditions of high cognitive demand. Dual-target HF-rTMS (left DLPFC + M1) has been shown to be more effective than single-target HF-rTMS (144). An RCT will compare the efficacy of dual-target anodal tDCS targeting the DLPFC and angular gyrus with that of single-target anodal tDCS of the DLPFC in PSCI patients, and will use fNIRS to investigate the underlying neural mechanisms (ChiCTR2500096896) (145). Another RCT will evaluate the efficacy of multi-target tACS applied to M1, primary sensory cortex, DLPFC, and cerebellum (ChiCTR2300073465) (146). Furthermore, Liu et al. (5) reported that stroke patients with concurrent cognitive and motor impairments exhibited hypoactivation in extensive sensorimotor and higher-order cognitive networks, while simultaneously demonstrating hyperactivation in the right prefrontal-parietal networks. Therefore, the right prefrontal-parietal network may represent a potential target for neuromodulation. These findings emphasize the necessity for a unified rehabilitation model that views cognitive and motor domains as dynamically interrelated, rather than isolated systems (147). We propose that multi-target stimulation should extend beyond cognition-specific targets to engage both physical movement and the neural circuits underlying cognitive-motor integration. Another application of multi-target neuromodulation is paired associative stimulation (PAS), an emerging technique that has been shown to enhance neuroplasticity and facilitate motor recovery after stroke (148). Nord et al. (149) investigated the effects of cortico-cortical PAS targeting the lateral prefrontal cortex and the intraparietal sulcus on decision making and working memory in healthy individuals. However, current evidence regarding PAS protocols for PSCI rehabilitation remains limited and warrants further investigation.

## 7 Multimodal strategies and holistic rehabilitation: clinical opportunities and emerging technologies

Different cognitive domains may require distinct optimal scalp stimulation protocols. LF-rTMS over the unaffected DLPFC may alleviate global severity, HF-rTMS over the left DLPFC may improve language, executive function, orientation, and attention deficits, and anodal tDCS over the affected DLPFC may enhance memory performance (150). Therefore, combining neuromodulation techniques exhibiting distinct functional effects with task-oriented training may provide more personalized and precise rehabilitation interventions for individuals with various cognitive impairments. Recovery of motor function after stroke is closely associated with executive function and attention (151). When patients optimize the allocation of attention and focus their limited cognitive resources on the most critical tasks, motor performance typically improves and fall risk is reduced. One such example is cognitive-motor dual-task training. Mou et al. (152) demonstrated that this approach increases the activation of PFC and the functional connectivity between motor and cognitive control regions, thereby improving motor function, walking ability, cognitive performance, and activities of daily living in stroke patients. Therefore, future research could consider combining cognitive enhancement-oriented neuromodulation with cognitive-motor dual-task training. Such multimodal interventions will achieve cognitive enhancement while concurrently improving motor function and supporting a hospital-home-community rehabilitation process, thereby advancing holistic rehabilitation after stroke.

Another promising direction for future development concerns combining the strengths of different neuromodulation strategies, including the integration of electromagnetic stimulation with ultrasound-based neuromodulation, chemogenetics, or BCI technologies. Hu et al. (153) demonstrated that bimodal rTMS-tDCS stimulation was more effective than single-modality stimulation in improving cognitive function in patients with post-stroke dysmnesia, suggesting a promising multimodal approach for the treatment of PSCI. It is noteworthy that combining neuromodulation with other neuroregenerative and neurorehabilitation approaches, such as stem cell transplantation, acupuncture, pharmacotherapy, and WM task, may also produce synergistic benefits (154).

## 8 Closed-loop neuromodulation based on brain-machine interaction: clinical implications of state-dependent mechanisms

Neuromodulation strategies hold significant potential for both cognitive enhancement and therapeutic interventions. However, a persistent challenge in the field is the considerable variability and inconsistency in findings across studies, often accompanied by small effect sizes. An important reason is the state-dependent mechanisms, which denote interactions between brain stimulation and prevailing neural network dynamics (155). For example, the network-level effects of TMS emerge from the interactions between an individual’s ongoing cognitive, emotional and/or perceptual brain state and the parameters of the external brain stimulation (156). Li et al. (157) used fMRI to investigate the brain state and polarity-dependent modulation of brain networks by tDCS. The results showed that, when applied at rest, anodal tDCS produced more pronounced effects than cathodal tDCS by increasing DMN activation and decreasing SN activation. During task performance, both anodal and cathodal tDCS increased SN activation, with cathodal stimulation producing a more pronounced effect. These findings highlight the importance of incorporating real-time physiological signal feedback into neuromodulation protocols to enable iterative adjustment of stimulation parameters. Closed-loop neuromodulation based on brain-machine interaction is a representative example of this concept.

Brain-machine interaction neuromodulation represents a leading frontier in BCIs. The transition from unidirectional brain-to-machine control to closed-loop, bidirectional brain-machine interaction via BCIs signifies a revolutionary breakthrough in neuromodulation. For patients with PSCI, closed-loop neuromodulation employs multiscale brain function detection (from cellular to systems levels), in conjunction with cognitive and behavioral assessments, to visualize and probe brain information. In closed-loop neuromodulation, acquired signals are decoded to adapt stimulation across modalities, categorized by stimulus type (electrical, magnetic, optical, or pharmacologic), spatial target (cellular structures, single neurons, or neural circuits), temporal resolution (from microseconds to minutes), and invasiveness (158). During neuromodulation, it is essential to emphasize the importance of enhancing rehabilitation motivation in stroke patients to establish the concept of “active health”. Our previous research has suggested that neurobiological changes after stroke may result in organic secondary apathy, which in turn increases the risk of post-stroke depression and cognitive impairment and reduces motivation for rehabilitation. Enhancing rehabilitation motivation among stroke survivors can promote active exercise and strengthen self-management, thereby mitigating physical impairment and facilitating overall functional recovery (159, 160). Therefore, reward strategies, task-oriented robotics, and virtual reality (VR) systems should be integrated with brain-machine interaction neuromodulation to normalize activity in relevant brain networks and strengthen patients’ autonomous engagement in rehabilitation. After neuromodulation, multimodal feedback (comprising visual, auditory, electrical, or haptic elements) is integrated to deliver neurofeedback and optimize stimulation parameters. Subsequent brain activity is recorded, and the loop iterates until precise control over the targeted neural circuits is achieved. Within this framework, future developments in brain-machine interaction neuromodulation will incorporate adaptive network-based modulation with predictive AI. Through brain-based and external sensors, these systems will automatically adjust and be controlled by cloud-based applications. The components will be introduced incrementally, ultimately culminating in a complete autonomous brain-stimulator-cloud interface (161).

Here, we illustrate an integrated closed-loop neurorehabilitation framework based on brain-machine interaction particularly for PSCI patients. Within this framework, most existing studies related to PSCI are derived from small-scale pilot studies using heterogeneous protocols, which limits the generalizability of the reported outcomes. However, the field is likely to continue moving toward the integration of AI in brain–machine interaction neuromodulation, which can dynamically adapt to the evolving needs of PSCI patients and enable personalized rehabilitation strategies.

## 9 Translational gaps and possible explanations

Most of the neuromodulation strategies discussed in this review are at different stages of clinical trials, while some novel technologies, such as TUS and genetic-based neuromodulation, remain at the preclinical stage. Overall, these strategies represent an emerging frontier for reducing the burden of PSCI, but numerous challenges still require further research to enable their broader application. First, the present landscape of neuromodulation for PSCI has largely evolved from studies primarily focused on ischemic rather than hemorrhagic stroke. Second, several studies have employed middle cerebral artery occlusion/reperfusion (MCAO/R) models, while many patients are unable to undergo urgent revascularization (162). Furthermore, animal models targeting other infarct regions, including deep penetrating vessels and posterior circulation, also require further investigation. Third, most current studies employ healthy adult rodents, whereas stroke primarily affects elderly individuals with comorbidities such as hypertension, atherosclerosis, and diabetes. Finally, the anatomical structure of rodents differs from that of humans. Therefore, future research should focus on developing animal-specific probes and coils or employing primate models to enhance the targeting precision and stimulation efficiency of neuromodulation, thereby improving the validity of preclinical evidence.

Several barriers to the clinical implementation of neuromodulation technologies also require significant consideration. The first barrier involves regulatory obstacles and reimbursement challenges. In the United States, recurrent courses of TMS are not commonly covered by insurance, and obtaining such coverage remains challenging (163). This absence of reimbursement creates a substantial financial barrier for patients. The second barrier is technical safety, which begins with ensuring that device design and manufacturing comply with applicable medical device safety standards and guidelines. The device maker should adhere to relevant national and international standards, evaluate the likelihood and severity of each potential risk, and implement appropriate measures to reduce each identified risk to an acceptable level (164). Regulatory authorities should also amend existing regulations to ensure that facilitate scientific progress without imposing unnecessary delays. The third involves increasing financial investment in neurorehabilitation infrastructure and providing rigorous technical training for therapists who perform neuromodulation. Finally, neuromodulation demands sustained user attention and engagement. Ensuring long-term adherence and usability in patients with cognitive impairment is both critical and challenging.

## 10 Conclusion and future prospects

A synthesis of preclinical and clinical evidence indicates that neuromodulation is associated with improvements in global cognition, memory, attention, language, executive function and visuospatial abilities in patients with PSCI. However, inadequate consideration of sample size, sham control quality, blinding integrity, and parameter heterogeneity may lead to an overestimation of the existing efficacy of certain neuromodulation techniques.

**Figure 3:**
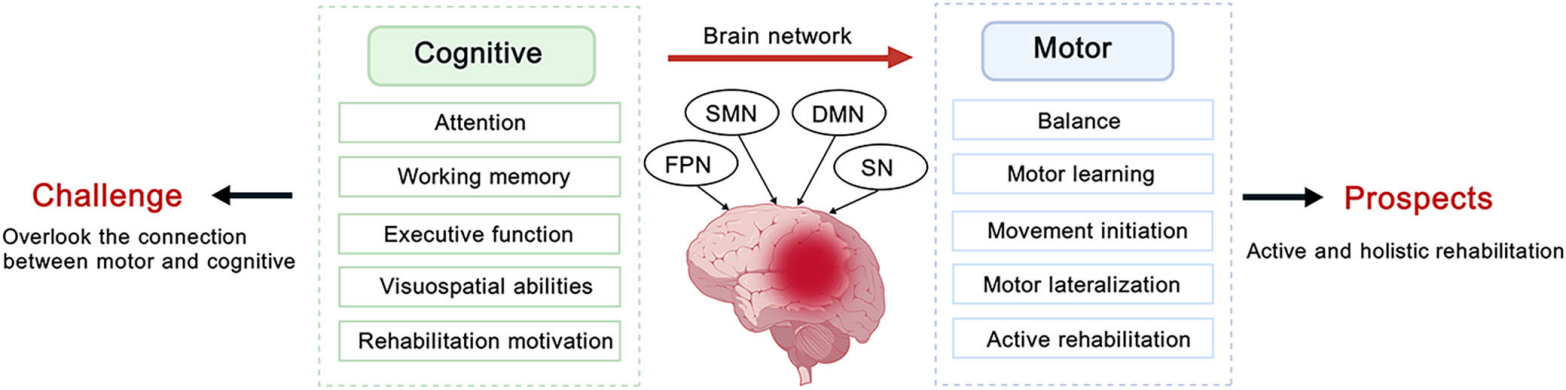
Future prospects of neuromodulation for stroke from the perspective of cognitive-motor interactions. The functional connectivity of the sensorimotor network (SMN), default mode network (DMN), frontoparietal network (FPN), and salience network (SN) in stroke patients mediates cognitive–motor interactions. We emphasize that future research should not focus solely on a single dysfunction following stroke, but rather consider the bidirectional facilitation between motor and cognitive functions to promote active and holistic rehabilitation. Figure created using BioGDP.com (7).

This review emphasizes that future clinical practice should consider the motor-cognitive interactions, implement multi-target neuromodulation, and investigate the temporal dynamics among stimulation targets to facilitate the reconstruction of impaired neural circuits. During rehabilitation, task-oriented approaches should be advocated to encourage active patient engagement, and neuromodulation strategies should be integrated with other neuroregenerative strategies to achieve multimodal interventions. Finally, neuromodulation is expected to advance toward brain-machine interaction neuromodulation, using AI to develop a closed-loop strategy encompassing stimulation, detection, optimization, and re-stimulation. Such a strategy aims to promote neural circuit reconstruction and enhancement, ultimately facilitating the overall functional recovery of stroke patients (**Figure 3**).
