# Supplementary material for: Neuromodulation and rehabilitation of post-stroke cognitive impairment: challenges and prospects
Source: Front Psychiatry. 2026 Feb 26;17:1780907. doi: 10.3389/fpsyt.2026.1780907 (PMC12979490; doi:10.3389/fpsyt.2026.1780907)
Supplement: Supplementary file 1 [file SupplementaryFile1.docx]

**Supplementary Material**

Multiscale brain function detection

Advances in multiscale brain function detection, ranging from the microscale to the macroscale, has undoubtedly revolutionized neurobiological research. At the microscale, brain function detection focuses on monitoring activity in individual neurons or small neuronal ensembles, thereby revealing alterations in the elementary units of neural information processing. These techniques primarily encompass microelectrode arrays and patch-clamp recordings. Furthermore, emerging photoacoustic modalities are included at this scale. For example, confocal microscopy produces high-contrast images with improved axial resolution for cellular and tissue imaging, and two-photon microscopy enables deep-tissue imaging with decreased photobleaching and phototoxicity, thereby rendering it well suited for studies of neuronal structure and dynamics^[1]^.

At the mesoscale, high-density silicon electrode arrays, such as Neuropixels probes, facilitate prolonged, simultaneous recordings from neuronal populations within defined cortical subdivisions within a single experiment^[2]^. Building on this foundation, Ye et al.^[3]^ developed Neuropixels Ultra (NP Ultra), which increases recording yield, enables detection of subcellular compartments, and improves cell-type identification, thereby facilitating a more effective dissection of neural circuit activity during behavior. Genetic-based neuromodulation, as exemplified by optogenetics and chemogenetics, enables the selective manipulation of defined cell populations with high spatiotemporal resolution, offering unprecedented opportunities to probe cellular mechanisms, trace neuronal projection pathways, and map neural profiles following neurological disorders^[4]^. Recent advances in genetic-based neuromodulation enable precise in vivo control of neuronal activity and circuit function. Consequently, these approaches offer new tools for monitoring post-stroke circuit dynamics. Functional ultrasound (fUS) employs Doppler ultrasound to detect neuronal activity and evoked changes in cerebral blood volume, making it well suited for systems-level interrogation of brain activity^[5]^. Combining optogenetic stimulation with fUS enables higher-sensitivity detection of evoked neural activity and reveals target-specific and circuit-level activation dynamics within specific cell types^[6]^.

At the macroscale, neuroimaging primarily captures whole-brain neural activity and characterizes the spatiotemporal dynamics of brain networks. A multimodal magnetic resonance imaging (MRI) approach, coupling diffusion tensor imaging (DTI)-based structural connectivity (SC) and resting-state functional MRI (rs-fMRI)-based FC, offers a more comprehensive understanding of the neurological characteristics and intrinsic correlation properties in stroke patients^[7]^. Cortical areas in PSCI patients exhibited lower FC-SC coupling in the occipital and frontal lobes compared to healthy individuals and stroke patients without cognitive impairment. Additionally, FC-SC coupling strength in the precuneus, paracentral lobule, and precentral gyrus correlates positively with cognitive performance. Three months after the stroke, increased FC–SC coupling strength was observed in the precentral gyrus and paracentral lobule, potentially reflecting an adaptive mechanism during cognitive recovery^[8]^. fNIRS research indicates that patients with PSCI show significantly reduced interhemispheric and intra-right hemispheric FC in comparison to healthy individuals, particularly in the somatosensory cortex, dorsolateral PFC, and medial PFC^[9]^. Magnetoencephalography (MEG) studies provide evidence that early post-stroke cognitive dysfunction has a network-level basis. During early recovery, interhemispheric connectivity from the ipsilesional to the contralesional cortex increases^[10]^. Finally, using machine learning models to analyze electroencephalography (EEG)-derived brain networks also help elucidate the neural mechanisms underlying stroke and recovery^[11]^.

[1] Ding C, Liu P, Xu Z, et al.Photoacoustic technologies in nervous system disorders: an emerging strategy for neuromodulation[J].Neural Regen Res,2025.

[2] Jun J J, Steinmetz N A, Siegle J H, et al.Fully integrated silicon probes for high-density recording of neural activity[J].Nature,2017, 551 (7679): 232–236.

[3] Ye Z, Shelton A M, Shaker J R, et al.Ultra-high-density Neuropixels probes improve detection and identification in neuronal recordings[J].Neuron,2025.

[4] He Y, Wei Z, Xu J, et al.Genetics-Based Targeting Strategies for Precise Neuromodulation[J].Adv Sci (Weinh),2025: e13817.

[5] Martinez De Paz J M, Macé E.Functional ultrasound imaging: A useful tool for functional connectomics?[J].Neuroimage,2021, 245: 118722.

[6] Brunner C, Grillet M, Sans-Dublanc A, et al.A Platform for Brain-wide Volumetric Functional Ultrasound Imaging and Analysis of Circuit Dynamics in Awake Mice[J].Neuron,2020, 108 (5): 861–875.e7.

[7] Liu X, Qiu S, Wang X, et al.Aberrant dynamic Functional-Structural connectivity coupling of Large-scale brain networks in poststroke motor dysfunction[J].Neuroimage Clin,2023, 37: 103332.

[8] Liu C, Zuo L, Li Z, et al.Brain structural-functional coupling mechanism in mild subcortical stroke and its relationship with cognition[J].Brain Res,2024, 1845: 149167.

[9] Zou J, Yin Y, Lin Z, et al.The analysis of brain functional connectivity of post-stroke cognitive impairment patients: an fNIRS study[J].Front Neurosci,2023, 17: 1168773.

[10] Soleimani B, Dallasta I, Das P, et al.Altered directional functional connectivity underlies post-stroke cognitive recovery[J].Brain Commun,2023, 5 (3): fcad149.

[11] Lee M, Hong Y, An S, et al.Machine learning-based prediction of post-stroke cognitive status using electroencephalography-derived brain network attributes[J].Front Aging Neurosci,2023, 15: 1238274.
